# Caregiver Perceptions of USDA Rural Non-Congregate Summer Meals for Children in California

**DOI:** 10.3390/nu18020270

**Published:** 2026-01-14

**Authors:** Emily Patten, J. Mitchell Vaterlaus, Lori A. Spruance, Christine Betty Crocker, Trevor Merritt, Lauren Wood

**Affiliations:** 1 Department of Nutrition, Dietetics and Food Science, Brigham Young University, Provo, UT 84062, USA; 2 Department of Human Development and Community Health, Montana State University, Bozeman, MT 59717, USA; j.vaterlaus@montana.edu; 3 Department of Public Health, Brigham Young University, Provo, UT 84602, USA; lori.spruance@byu.edu; 4 Lodi Unified School District, Lodi, CA 95240, USA; bcrocker@lodiusd.net

**Keywords:** school nutrition, school meals, summer feeding, rural non-congregate meals, food insecurity

## Abstract

**Background/Objectives:** In 2023, the United States Congress amended Section 13 of the National School Lunch Act to allow non-congregate meal service as an option within the Summer Food Service Program in rural areas, creating “SUN Meals To-Go.” The purpose of this qualitative study was to explore caregivers’ perceptions of USDA rural non-congregate summer meal programs in California during the summer of 2024. **Methods:** This was a cross-sectional, qualitative study using an electronic 20-item survey instrument that was available in English and Spanish. Five school foodservice directors in California shared and/or posted at meal pick-up sites a flyer with a QR code leading caregivers to the survey instrument. A conventional content analysis was conducted with the open-ended responses and descriptive statistics were calculated for close-ended items. **Results:** Caregivers (n = 827) were primarily married (70.5%) and Hispanic/Latino (54.3%) women (85.5%). They (55%) reported using the 2024 summer meal program “most times” or “every time” it was available. Three themes were constructed through qualitative content analysis: (1) Family support and resource relief, (2) Navigating program accessibility and logistics, and (3) Nourishment and practicality: Reflections on food quality, nutrition, and sustainability. **Conclusions:** Caregivers highlighted that the program supported their families and provided resource relief. They indicated that accessibility and logistics were effective, provided ideas for fine-tuning the delivery of the program, described this program as supporting their children’s nutrition.

## 1. Introduction

Food insecurity is a national issue, affecting 17.9% of households with children in 2023 [1]. In California specifically, 56.2% of school aged children’s families experienced food insecurity [2]. Food insecurity is associated with increased prevalence of anxiety, depression, nutritional deficiencies, delayed cognitive development, behavioral problems, and overall poorer health [3]. During the summer, when school are closed and meals are unavailable, households with children experience greater rates of food insecurity [4]. To address this concern, the United States Department of Agriculture (USDA) offers several summer nutrition programs for children [5]. Participation in these programs is associated with lower risk of food insecurity [6]. However, food insecurity remains common in rural areas due to lack of sites, transportation issues, and overall reduced accessibility [7,8].

Until recently, children of any age could go to a summer meal site at a school, park, or other location and consume a meal and/or snack [9]. In 2023, the United States Congress amended Section 13 of the National School Lunch Act to allow non-congregate meal service as an option within the Summer Food Service Program (SFSP) and Seamless Summer Option (SSO) in rural areas, creating “SUN Meals To-Go” [10,11]. In the spring of 2024, the USDA’s Food and Nutrition Service (FNS) Office of Policy Support surveyed sponsors of these 2023 summer non-congregate meal service sites to evaluate program perceptions and processes [12]. Ultimately, sponsors reported they were able to provide more meals to children at a cost less than or equal to the cost of congregate meals. Sponsors primarily offered these meals through pick-up (79%) or a combination of pick-up and delivery (15%). Additionally, sponsors reported allowing parent/guardian pick-up on behalf of participants (74%), issuance of multiple days’ worth of meals (59%; 55% of these provided 5–7 days’ worth of meals at one time), and distribution of bulk food (32%). Furthermore, most sponsors intended to sponsor meals again that summer (86%) and 19% planned to expand their services by either adding more sites and/or providing more meals [12].

Following the inaugural program summer in 2023, *Share Our Strength* interviewed 30 and surveyed an additional 10 caregivers of participants in the rural non-congregate feeding program across three states to gain their insight about the program [13]. They found caregivers described universal financial relief for their families as the meals freed up money that would have normally been allocated to their food budget. Overall, *Share Our Strength* concluded that the 2023 rural non-congregate meal program was effective and helped reach more children in rural communities and recommended expanding the program [13].

While these early evaluations provide important initial insights regarding benefits of the rural non-congregate meal programs, they are limited. Existing assessments reflect sponsor or organizational perspectives and were published in non-peer-reviewed formats. To date, there are no peer-reviewed research studies focused on caregivers’ experiences with non-congregate summer meal programs in rural areas. Furthermore, little is known about how families in diverse areas perceive this program type. Addressing this gap is essential for informing policy and enhancing program implementation as this program continues to expand. Accordingly, the purpose of this qualitative study was to explore caregivers’ perceptions of rural non-congregate summer meal programs in California during the summer of 2024, with the goal of informing broader policy and day-to-day practice.

## 2. Materials and Methods

**Study Design and Survey Instrument.** This cross-sectional study employed a qualitative design with descriptive quantitative data used for triangulation [14]. Quantitative descriptive statistics were used to contextualize and corroborate qualitative themes, not for inferential purposes.

Consistent with qualitative research design guidance, the survey items were developed in alignment with the research problem and the existing literature that inform it [14]. The literature review focused on summer meal programs [15,16,17,18] and bulk school meal distribution during the COVID-19 pandemic [19,20,21,22]. The survey instrument was developed by one academic researcher and then revised and edited by other research team members including two other academic researchers with experience researching school nutrition topics and a school district foodservice director (doctorally trained). The qualitative open-ended items were purposefully written to be open, short and as unambiguous as possible [23]. The survey was designed to have a coherent flow, and it closed with a final open-ended item to assure respondents could share any unanticipated or useful data that may not have been gathered through the other items [23]. Once finalized in English, the survey instrument was professionally translated into Spanish by an authorized translator through Connected Translation LLC (Reno, NV, USA). The Spanish version of the survey was also reviewed for clarity by two Spanish speakers familiar with school meal programs and no changes were made.

The survey had 20 items. The first item asked participants in both English and Spanish which language they preferred and then it branched to either an English or Spanish version of the survey. Five items invited open-ended responses about caregivers’ thoughts and experiences with the program; one of those items was only presented to those who indicated they did not participate in the program every time it was available. One matrix item had a series of 20 statements about the program for which respondents rated their agreement on a 5-point scale, later used to triangulate the qualitative data. Personal and household characteristics were also queried. Brigham Young University’s IRB deemed this study exempt prior to data collection (IRB2024-242). The sponsoring institution did not have any influence on the survey design or content. Participants were presented with a consent statement in their preferred language (English or Spanish) and then implied their consent by completing the survey.

**Data Collection.** Through convenience sampling, five school foodservice directors were recruited in 2024 to distribute the survey instrument to the caregivers participating in their summer meal program. The five school foodservice directors in northern (n = 1) and southern (n = 4) California shared and/or posted at meal pick-up sites a flyer with a QR code leading caregivers to the survey instrument. The flyer had information about the survey in both English and Spanish. At the end of the survey, respondents were invited to a separate survey instrument to enter a drawing for one of twenty $25 Amazon gift cards.

In total, 1667 surveys were initiated, and of those, 827 were usable. Responses were removed if they did not complete through the matrix agreement scale item or did not have an appropriate reCAPTCHA score (scores < 0.5 were removed). The Spanish open-ended responses were professionally translated from Spanish to English by an authorized translator through Connected Translation LLC and then the English (n = 748) and Spanish (n = 78) survey responses were combined into one dataset.

**Data Analysis.** The five open-ended items analyzed include the following: (1) Why did you not participate in all of the available To-Go meal services? (2) In what ways has the school district offering To-Go meals influenced you and your family this summer? (3) Please write any comments you might have regarding your level of agreement to the previous statements here (this item is related to the matrix agreement item). (4) What are any benefits or challenges you have experienced with the school district’s To-Go meals this summer? (5) Is there anything else you would like share about your experience with the To-Go meals this summer?

A conventional qualitative content analysis approach was used to analyze the data, which is an inductive technique [24]. Consistent with the approach, four researchers immersed themselves in the data by reading and rereading responses. Next, they met to discuss their thoughts and disclose any reactions or potential biases they had about the data. Together, they developed a codebook by identifying and defining codes. Next, two of those researchers independently coded the data set. Initially, they coded subsets as they refined the code book, and then ultimately, they coded the entire data set using 20 defined codes. Interrater agreement was 83.5%. The two researchers who completed the coding coordinated with each other to resolve coding discrepancies and after discussion they created and agreed upon a final coded dataset. Finally, the same four researchers met again to organize the codes into three themes capturing the collective experiences and perspectives of respondents.

Descriptive statistics were calculated for all the variables using SPSS (version 29). To triangulate the qualitative data, means were calculated for responses to 20 scaled items assessing caregivers’ experiences with and perspectives on the summer to-go program. These responses were on a 5-point scale (1 = strongly disagree, 5 = strongly agree); therefore, higher means indicate greater agreement. After the qualitative analysis was completed, the researchers reached consensus on how and where to report descriptive results from the fixed-response items within the qualitative theme narrative to contextualize and corroborate the qualitative themes.

## 3. Results

In total, there were 827 usable responses. Respondents were primarily married (70.5%) and Hispanic/Latino (54.3%) women (85.5%) (See Table 1). There was representation across the range of possible household incomes presented (<$10,000/year to >$150,000/year). Respondents (55%) reported using the 2024 summer to-go meal service “most times” or “every time.”

Three themes were constructed through qualitative content analysis: (1) Family support and resource relief, (2) Navigating program accessibility and logistics, (3) Nourishment and practicality: Reflections on food quality, nutrition, and sustainability (see Table 2). Themes are presented in order of prevalence. When direct quotes are shared, participant gender, age, and race/ethnicity are provided parenthetically for context. Table 3 presents all the means for respondents’ reported degree of agreement on scaled survey items.

## 4. Theme 1. Family Support and Resource Relief

Caregivers (41%) expressed deep gratitude for the to-go meals program, calling it a “blessing,” “support,” “help,” “supplementation,” and “assistance.” While appreciative, many elaborated on how the program specifically supported their families. A participant shared, “Well, [the to-go meals were] a great help. Nowadays, the market is very expensive, and we don’t have enough money, and the help from the district puts food on our table. Thank you!” (53 years, female, White, Hispanic). Consistent with these statements, caregivers largely agreed with the fixed-response item: “To-Go meals helped my family financially” (*M* = 4.47 ± 1.04). Caregivers recognized that feeding their children was their responsibility—“It is not the district’s responsibility to feed my children, but we are deeply grateful to them for doing so” (37 years, female, White, Hispanic). In line with that, the fixed-response item “It is not the school district’s responsibility to feed my child(ren) during the summer” had a mean of 3.69 ± 1.23. Caregivers felt the burden of economic hardship, food insecurity, and time constraints. The statement “Without To-Go meals, my child(ren) would not have enough food to eat during the week” had a mean of 3.05 ± 1.42 which is near the “neither agree nor disagree” scoring level. Several caregivers acknowledged that the program helped them fulfill their responsibility of feeding their own children, while expressing deep appreciation for the district’s support.

Beyond food provision, 52.7% of caregivers shared that the program positively impacted other resources, including time. A participant explained that

*I was able to spend more time with my kiddos since the meals were prepped already. I didn’t have to spend so much time in the kitchen or washing up. Took my kids out to do the city activities and other outings.* (26 years, female, Hispanic)

Appreciation was expressed for the time saved on meal preparation that allowed caregivers to dedicate more time to their children. Caregivers (8.5%) who worked during the summer expressed relief knowing their children—especially those home alone—had access to food that they could self-prepare. For instance, a participant shared the following:

*To-go meals helped tremendously. We run out of food about 3 weeks into each month during school and have to struggle the rest of the month and sometimes school breakfast and lunch are the only meals the kids get. So, I was very worried when summer got here but the schools to-go meals relieved so much stress and it also helped relieve the stress from the kids making their own food while I’m at work.* (46 years, female, White)

Others reported that the program freed up money for school-related expenses, such as clothing and supplies. A participant stated, “… [I am] extremely grateful they [provided this program]. The district helping feed my kids left me enough money to buy back to school clothes, shoes and back to school supplies” (52 years, female, White, Hispanic). Having food support was seen by caregivers as a way to meet their children’s broader needs. Additionally, 6.9% of caregivers noted that meal pick-up became a “fun” activity for their children, particularly when located near parks or libraries.

In sum, while caregivers affirmed their responsibility to feed their children, they expressed sincere gratitude for the to-go meals program. It not only provided essential nourishment but also extended limited family resources—particularly time and money—enabling caregivers to meet broader family needs. Further, most caregivers indicated agreement with the statement: “my family would participate in this program again next summer” (*M =* 4.66 ± 0.81).

## 5. Theme 2. Navigating Program Accessibility and Logistics

Caregivers addressed their experiences with the implementation aspects of the to-go meals program. Several caregivers (10.6%) expressed appreciation for the staff who distributed meals. One participant shared, “The servicing staff was very kind and respectful even when things [were] chaotic the first day. I appreciate them” (74 years, female, Black). Staff were frequently described as “helpful,” “kind,” “friendly,” “quick on their feet,” and as individuals who “always had a smile.” Open-ended comments were congruent with caregivers’ agreement with the statement “the staff at the To-Go meal pick up were helpful” (*M* = 4.70 ± 0.79).

Overall, the fixed-response items relevant to program accessibility and logistics were highly rated by caregivers (see Table 3). However, caregivers identified areas where program implementation could be improved through open-ended responses. A portion (12.4%) reported unclear communication at the start of the summer, with caregivers stating things like, “I did not know about it until after it started” (36 years, female, White). While some acknowledged they received emails, they did not read them closely or were unable to locate key details about time and location. As the summer progressed, many learned about the program through neighbors, friends, or school text messages. These caregivers expressed a desire for earlier and clearer communication to maximize the program’s benefits.

Logistical challenges related to pick-up schedules (38.6%) and location (6.4%) were also cited. Early in the program, long lines and extreme temperatures created barriers. Some caregivers waited in the line for a long time to receive food, but had to leave to meet work, family, and other personal appointments (e.g., “[The] long line was hard. [I] would wait in line an hour but had to leave for work.” [37 years, female, Asian]) or they did not receive food because the program ran out. Others noted that distant pick-up sites or lack of transportation limited their ability to participate, with comments like, “did not have a car” or “did not have a ride.”

Considering these challenges, many caregivers offered practical suggestions to improve accessibility and efficiency (9.2%). A participant proposed, “I would like something closer to home and [possibly] a few days out of the week for pick up versus one day for everyone that causes congestion and long lines/wait times” (26 years, female, Black, Hispanic). Recommendations included offering pick-up sites near children’s schools, varying pick-up times and days to accommodate work schedules, and improving line management. Some also advocated for more flexible rules, such as not requiring children to be present or allowing caregivers to collect meals for other families.

Despite the logistical challenges, caregivers viewed the to-go format as a significant improvement over previous summer meals programs. A caregiver reflected,

*I loved the convenience of picking up meals for the whole week. When my children were younger, they had to eat at the park, it was so hot outside, and they even had to sit where the food was distributed and not on the benches where us parents were. It felt like a prison; students couldn’t even take their meal home. The school lunch staff would tell them to throw it away once they got up off their seats. This time around picking up meals, the kids being able to eat in the comfort of their home and not outside in the heat THIS WAS THE BEST CHANGE EVER! I wish it was implemented 10 years ago, but hey someone smart within the food service school district program finally used their common sense on better serving the community. Thank you!* (40 years, female, Hispanic)

Caregivers emphasized that, while improvements were needed, the program’s core approach was effective and highly valued. Many expressed a strong desire for its continuation in future summers, believing that small adjustments could make a good program even better.

## 6. Theme 3. Nourishment and Practicality: Reflections on Food Quality, Nutrition, and Sustainability

Caregivers reflected on the quality, nutritional value, and usability of the meals provided through the to-go meal program. A common point of appreciation in open-ended responses was the inclusion of fresh fruits and vegetables (15.6%), which many families indicated had a meaningful impact on their eating habits. Caregivers largely agreed that “To-Go meals increased the number of fruits and vegetables my child(ren) ate this summer” (*M* = 4.20 ± 1.12). In an open-ended response a caregiver shared, “Having fresh fruits and vegetables helped us eat more fresh healthy foods. I never had a fresh pineapple before this program” (42 years, female, Black). Others noted that the regular availability of fresh produce encouraged more balanced (18.3%), home-prepared meals (e.g., “It helped us to make more meals at home.” [45 years, female, American Indian or Alaskan Native]).

In addition to praising fresh items, participants highlighted how the meals supported their children’s nutrition. Some mentioned that the inclusion of protein-rich items contributed to meeting their children’s dietary needs. However, there was also feedback expressing a desire for more variety in the protein options (e.g., “Some days, I wished for more protein options” [46 years, male, White, Hispanic]). Concerns were also raised about the milk offerings, particularly regarding expiration and lack of alternatives: “The milk expires too early and there was no option for vegetarian for pick up and [no] milk substitute or lactose free milk” (41 years, female, White). A few caregivers also requested more food choices to address allergies or religious/cultural needs (e.g., Halal, Kosher).

While caregivers indicated agreement with the statements “my child(ren) enjoyed the food from the To-Go meals” (*M =* 4.43 ± 0.90), “the To-Go meals provided high quality food” (*M* = 4.27 ± 0.96), and “To-Go meals increased the variety of foods my child(ren) ate this summer” (*M* = 4.2 ± 1.12), a recurring suggestion involved increasing the variety of meals overall. A few caregivers (3.4%) expressed that while their children initially enjoyed the offerings, repetition diminished enthusiasm. A caregiver explained,

*While I am so so SO grateful for these opportunities for my children … I do think different varieties of meals would be great! They loved all the different snacks and fruits that were given but did get a little bored of the same lunches.* (35 years, female, White)

Most caregivers indicated agreement with the statement “my family knew how to prepare the food that we were provided from To-Go meals” (*M* = 4.39 ± 1.07), but there was some open-ended feedback that touched on the challenges of food preparation and sustainability—particularly regarding frozen items. While frozen meals offered convenience, several caregivers were concerned about the lack of preparation instructions, which created food safety and usability issues. A participant described,

*I was disappointed in the amount of frozen items included. None of the frozen items included instructions on how to prepare them. How long to heat? Whether to use the oven/microwave, stovetop? It created a safety issue not knowing how long was too long [to heat] and if the items would burn when trying to pick them up or be inedible from undercooking or overcooking. Unfortunately, several of the burritos, tacos, mac n cheese and other frozen items were disposed of due to no instructions provided.* (37 years, female, Hispanic)

Some caregivers acknowledged that food quality concerns may have stemmed more from children’s preferences than from the meals themselves. Others raised considerations about sustainability, including both food waste and packaging. A participant commented, “I’m concerned about the environmental impact of excessive packaging” (40 years, White, Black, Hispanic), while others appreciated the eco-friendliness of the materials and felt the packaging reduced waste compared to takeout containers.

Most caregivers indicated agreement with the following fixed-response items: “we had enough refrigerator space to store the To-Go meals at our home” (*M =* 4.47 ± 0.87), “we had enough freezer space to store the To-Go meals at our home” (*M =* 4.33 ± 1.02), and “we had enough counter and/or cupboard space to store shelf-stable foods from To-Go meals at our home” (*M =* 4.53 ± 0.86). However, in open-ended items, a few participants noted challenges related to food storage, especially when receiving large quantities at once. A participant shared, “[It is a challenge] finding space to store all the milk” (36 years, female, American Indian or Alaskan Native, Native Hawaiian or Pacific Islander, Hispanic), emphasizing how the volume of distributed food could strain household storage capacity. Notably, caregivers disagreed that “receiving fewer meals at a time would work better for my family” (*M* = 2.08 ± 1.32) and “there was a lot of food wasted from the To-Go meals” (*M* = 1.99 ± 1.21). Overall, while families valued the nutritional benefits and home-use flexibility of the to-go meals, they also provided thoughtful suggestions related to variety, preparation instructions, and sustainable practices.

## 7. Discussion

Overall, responding caregivers indicated the USDA rural non-congregate meal program was positive for their families and they highly rated their experiences with the program. Caregivers described this program as being supportive of their families and as a source of resource relief. In the USA, consumer prices have risen 2.4% from May 2024 to May 2025 and food prices have gone up 2.9% [25]. When compared across states in the USA, California has the highest regional price parities (RPP), which measure the differences in prices across the USA; California is at 112.6% with the overall national price level being 100% [26]. Historically, research has shown that food insecurity rises in the summer for children from lower-income homes who participate in the National School Lunch Program [27,28,29]. While there was lower agreement with the statement “Without To-Go meals, my child(ren) would not have enough food to eat during the week” (*M* = 3.05 ± 1.42), the program likely supplemented other household resources during the summer months. Caregivers in this study did indicate that this targeted program helped their families financially and saved them time. These findings align with prior research on families of school-aged children. Zuercher et al. [2] found that Californian parents with and without food insecurity similarly reported school meals saving them time and money. Likewise, in a study of parents’ perceptions of universal school meals, parents identified conservation of family resources (time and money) as a key benefit [30]. Overall, nutrition programs appear to provide meaningful support to families.

Responding caregivers highly rated most elements of the program and their experiences with it via the fixed response items, indicating that overall, they were pleased with the program delivery. Through their open-ended responses they provided specific feedback for improvement that these and other non-congregate meal sites may consider in their summer meals programs. One area of improvement was that respondents desired earlier and clearer communication about the program. In a study about summer meals during COVID-19, respondents reported lack of awareness of the program as a barrier and expressed the desire for receiving communication regarding summer meals from their child’s school [31]. In another study, the most common reason parents gave for not participating in summer meals programs was that they were not aware of sites near their homes [32]. As this program type becomes more ubiquitous, word of mouth across the community may help resolve this concern. However, if initiating this program, foodservice directors should consider their communication plan including timing and modes of communication with caregivers.

Other caregiver feedback focused on the logistics of picking up meals. Some respondents found the schedules and locations to be barriers to their use of the program. Respondents highlighted long wait times/lines and some challenges with having limited days/times for meal pick-ups. As directors interact with participants in their communities and gain more experience with the program, there may be creative solutions to these challenges. Similar challenges were faced during the COVID-19 pandemic when school meal programs had to adjust their service models. In one study of foodservice directors in a northeastern state, waivers allowed flexibility in the new rural non-congregate summer meal program [15]. In that study, one director extended meal service from 3:00 to 6:00 p.m. to accommodate parent work schedules, others utilized bus routes through town to enhance public access, and one director coordinated with the transportation department to map town routes that would keep walking time to a pick-up site within 10 min [15]. In another study, a school district in California adjusted their service model to accommodate families, leading to substantial organizational changes [33]. As a result, meals increased by 147% during the pandemic in their district, whereas across the nation they declined 30% [33]. As school districts and other sponsors are seeking to tailor their summer meals programs to work for their community, there may also be opportunities to partner with nearby universities to have business, dietetics, public health, and/or hospitality students/faculty help assess and identify opportunities to increase efficiency within the constraints of the program and in the context of the community [34]. These examples, among other creative, individualized solutions, may be leveraged to increase accessibility.

Caregivers in this study indicated that their children ate a variety of foods, more fruits and vegetables, and had more home-prepared meals due to this program. Recent research investigating the foods and nutrients offered in the other USDA summer meals programs (summer food service program and seamless summer option) has shown that overall, summer lunches substantially contribute towards meeting Dietary Guidelines for Americans’ nutritional goals for 9–13-year-old children [35]. This is vital given that most children this age do not meet daily fruit and vegetable recommendations [36]. Future research could include this new program type to further explore the nutritional impact of USDA programs.

Caregivers’ agreement with statements about number of meals provided being appropriate and having adequate home storage space for the meals (fridge, freezer, counter/cupboard)—paired with their disagreement with statements about preferring to receive fewer meals and that there was a lot of food waste—demonstrates that this program offers an efficient solution to families. While open-ended responses showed that sometimes there were challenges like having too much milk or not knowing how to prepare/heat a food item, the overall agreement means for these program elements were high. As foodservice directors continue to implement these programs, applying methods for process improvement may help them refine how the programs are delivered to children and their families [37].

**Practical Implications.** Considering the rise in food prices and variation in cost of living across the USA, summer meal programs may play a more important role in food budgeting for families, even if it is not the primary food source. For those organizing summer meal programming, it is important to have early, clear, and repeated communication to reach families. Additionally, finding ways to increase logistical flexibility (e.g., adjusting pick up times or days) and continuing to collect caregiver feedback for continuous improvement may increase the likelihood of family participation. For those involved in developing policy and conducting research, caregivers in the current study reported their children had more variety of foods and especially that they ate more fruits and vegetables due to this program. Further research, along with leveraging those findings to support continued funding, could be useful for mitigating food insecurity and optimizing summer nutrition for children.

**Limitations.** There are limitations to this study that should lead to caution in generalizing these findings beyond this sample. First, this is a qualitative study focused on one geographic area in the USA. Additionally, the use of convenience sampling is a limitation because participants were likely from engaged families who were willing to join the study. Further, the sample included high representation from the Lodi Unified School District which may also limit the relevance of these findings to other school districts. This study focused only on caregivers who participated in the program and did not include perspectives of those who opted out. Finally, this study was constrained to caregivers’ perspectives and future research should explore children’s experiences with the program.

## 8. Conclusions

This qualitative study of caregivers’ experiences and perspectives on rural non-congregate summer meals programs in California indicates that this sample found value in and appreciated the program. Caregivers in this study highlighted that the program supported their families and provided resource relief. They indicated that accessibility and logistics were effective and provided ideas for fine-tuning the delivery of the program. Finally, caregivers in this study described this program as supporting their children’s nutrition.

## Figures and Tables

**Table 1:** Characteristics of responding caregivers (n = 827).

Characteristic	Mean	SD
Age (years)	39.83	8.02
	n	%
Gender		
Male	108	13.1
Female	707	85.5
Non-binary/third gender	2	0.2
Prefer not to say/Did not report	10	1.2
Race ^a^		
White	497	60.1
Black or African American	37	4.5
American Indian or Alaskan Native	13	1.6
Asian	67	8.1
Native Hawaiian or Pacific Islander	3	0.4
Other	159	19.2
Prefer not to say	80	9.7
Hispanic, Latino, or Spanish origin		
Yes	449	54.3
No	333	40.3
Prefer not to say/Did not report	45	5.2
Marital Status		
Married/Partnered	583	70.5
Widowed	11	1.3
Divorced	58	7.0
Separated	29	3.5
Never Married	109	13.2
Prefer not to say/Did not report	37	4.5
Household Annual Income		
<$10,000	59	7.1
$10,000–$19,999	59	7.1
$20,000–$29,999	74	8.9
$30,000–$39,999	75	9.1
$40,000–$49,999	92	11.1
$50,000–$59,999	68	8.2
$60,000–$69,999	76	9.2
$70,000–$79,999	59	7.1
$80,000–$89,999	65	7.9
$90,000–$99,999	43	5.2
$100,000–$149,999	61	7.4
>$150,000	20	2.4
Prefer not to say/Did not report	76	9.2
School District Sponsoring the Meal Program		
Lodi Unified School District, Northern California, USA	366	44.1
Rialto Unified School District, Southern California, USA	18	2.2
Val Verde Unified School District, Southern California, USA	205	24.7
Needles Unified School District, Southern California, USA	28	3.4
Morongo Unified School District, Southern California, USA	191	23.0
Other/Did not report	21	2.5
Family frequency of participating in 2024 summer’s to-go meal service		
One time	114	13.8
A few times	257	31.1
Most times	236	28.5
Every time	219	26.5
Did not report	1	0.1

^a^ Participants could select as many race classifications as desired, for this reason the total percent exceeds 100%.

**Table 2:** Caregiver (n = 827) perspectives on rural to-go meals: Results from a qualitative content analysis.

Codes	n	%	Representative Quotations
Theme 1: Family Support and Resource Relief			
Family Resources	436	52.7	“It helped us because I didn’t have much money for food.” (37 years, female, Hispanic) “[The to-go meal program] helped give my kids meals all summer when we are struggling at the moment.” (34 years, female, White, Hispanic)
Gratitude	343	41.5	“Thank you for providing to go meals. Really helped us stretch our dollar to feed all of the kids in this economy all summer long. It was appreciated!” (46 years, female, White) “It was like Christmas to the kids [when we picked up food]. … We are so very grateful! Thank you!” (67 years, female, White)
Provides Food for Children While Parent is Working	70	8.5	“To-Go meals helped tremendously. We run out of food about 3 weeks into each month during school and have to struggle the rest of the month. Sometimes school breakfast and lunch are the only meals the kids get. So, I was very worried when summer got here but the schools To-Go meals relieved so much stress, and it also helped relieve the stress from the kids making their own food while I’m at work.” (46 years, female, White) “[My daughter] is at the age where she can stay at home [while I’m at work] which helps with not having childcare but having her at home increases groceries. The food [from the to-go program] helped!” (32 years, female)
Activity to do with Children	57	6.9	“It brought smiles and excitement to my children’s faces knowing we were going to pick up meals every time.” (32 years, female, Mexican American) “It was fun, convenient and easily accessible at the library.” (42 years, female, Asian, Hispanic)
Theme 2: Navigating Program Accessibility and Logistics			
Program Scheduling Conflicts	319	38.6	“Long line, would wait in line an hour but had to leave for work.” (29 years, female, White) “Because the lines were very long and there was no time to wait so long because of work.” (40 years, female, Mexican)
Program Communication	102	12.3	“I did not know about [the to go meals program] until after it started.” (36 years, female, White) “The memo [about the to-go meals program] was sent from my friend. I never received any information about it.” (45 years, female, White, Hispanic)
Staff Were Great	88	10.6	“The volunteers or the to-go meal staff were very friendly, respectful and very helpful! They interacted with my child in the vehicle and made her smile and laugh at times even if she’s a bit older, they still made time to interact with her. I truly appreciate that! And you have to commend them for working out in the desert heat during the summer to help families.” (32 years, female, Asian) “Overall, thank you for the help, any help is always appreciated of course and thank you to all the friendly helpful staff members who helped us. I strongly suggest they all receive raises!” (40 years, male, White)
Opportunities to Improve Efficiency	76	9.2	“It will be nice to have different times to pick up, especially in the afternoon. Some parents are at work during the give out. That is one of the reasons we were not able to participate all the days of the give out.” (28 years, female) “Maybe make smoother pickups or multiple locations. First time I went [to pick up food] I waited in line for over 2 h.” (39 years, female, Hispanic)
Location Challenge	53	6.4	“Having a more convenient location would be helpful for next summer!!! Driving distance was too far, but the service provided made it worth it.” (42 years, female, Hispanic) “I wish [the pick-up location] had been closer to the school where my children attend since I had to drive 25 min to pick up food and 25 to come back.” (43 years, female, Hispanic)
Transportation	37	4.5	“[I missed some of the pick-ups because I] Couldn’t make it in time and didn’t have a ride.” (38 years, female, Hispanic) “[We missed because of lack of] transportation, and it was really too hot for the kids to walk.” (38 years, female, Hispanic)
Theme 3: Nourishment and Practicality: Reflections on Food Quality, Nutrition, and Sustainability			
Balanced and Healthy Meals	151	18.3	“It was a big help to our household. Definitely helped make sure our kids had a balanced and healthy meal.” (46 years, female, White, Hispanic) “We made it a priority to cook at home more often [with the to-go meals] instead of dining out.” (36 years, female, Hispanic)
Fresh Fruits and Vegetables	129	15.6	“The produce was amazing, my children tried foods they wouldn’t normally try.” (46 years, female, White) “We especially loved the fresh fruits/vegetables since that’s usually something out of budget for us.” (29 years, female, White)
Packaging and Sustainability	44	5.3	“The packaging was environmentally friendly, which I liked.” (43 years, male, White, Hispanic) “The frozen food was not very good. There were somethings in the snack packages that were not very appetizing. … the cereal and milk were appreciated. The Mexican food in the frozen bag was terrible.” (72 years, female, White) “Because the frozen food was in large bulk packages, it was difficult to find freezer space for everything. Also, we had to defrost and eat the food in the large packages day after day in order to avoid waste. Smaller packages of the frozen foods would be helpful.” (57 years, female, Asian)
Food Quality and Preparation	43	5.2	“Frozen and shelf stable food much less healthy than fresh. Would like to see more fresh fruits/veggies and canned goods and staples like pasta or rice rather than the cheap frozen meals like pizza and tacos.” (46 years, female, White) “It would be helpful if the frozen food came heating or cooking instructions.” (45 years, male, Hispanic) “The frozen packaged food was unappetizing. Prefer fresh vegetables and fruit.” (43 years, White, Hispanic)
Limited Variety in Food Choices	28	3.4	“My only concern it was the same meals every other weekend.” (41 years, female, White, Hispanic) “We kept getting some of the same food over and over.” (40 years, male, White) “My kids get tired of the limited choices.” (50 years, female, White)

Notes. Percentages exceed 100% because participants could contribute to multiple codes. Participant age, gender, and race/ethnicity were provided parenthetically after quotes to provide context.

**Table 3:** Caregiver agreement with statements about rural non-congregate meal programs (“to-go meals”) as they align with themes from the qualitative analysis of open-ended responses (n = 827).

	*M* ^a^	SD
Theme 1: Family Support and Resource Relief		
My family would participate in this program again next summer.	4.66	0.81
To-Go meals helped my family financially.	4.47	1.04
It is not the school district’s responsibility to feed my child(ren) during the summer.	3.69	1.23
Without To-Go meals, my child(ren) would not have enough food to eat during the week.	3.05	1.42
Theme 2. Navigating Program Accessibility and Logistics		
The staff at the To-Go meal pick up were helpful.	4.70	0.79
It is better for my family to pick up multiple To-Go meals rather than bringing my children to receive a meal each day.	4.60	0.94
The process of getting my child(ren)’s meals was efficient.	4.33	1.15
The pick-up times worked for my family.	4.21	1.16
The pick-up location was convenient for me.	4.12	1.28
Theme 3. Nourishment and Practicality: Reflections on Food Quality, Nutrition, and Sustainability		
We had enough counter and/or cupboard space to store shelf-stable foods from To-Go meals at our home.	4.53	0.86
We had enough refrigerator space to store the To-Go meals at our home.	4.47	0.87
My child(ren) enjoyed the food from the To-Go meals.	4.43	0.9
My family knew how to prepare the food that we were provided from To-Go meals.	4.39	1.07
We had enough freezer space to store the To-Go meals at our home.	4.33	1.02
The number of To-Go meals provided was just right for my child(ren).	4.27	1.06
The To-Go meals provided high quality food.	4.27	0.96
To-Go meals increased the variety of foods my child(ren) at this summer.	4.21	1.12
To-Go meals increased the number of fruits and vegetables my child(ren) ate this summer.	4.20	1.12
Receiving fewer meals at a time would work better for my family.	2.08	1.32
There was a lot of food wasted from the To-Go meals.	1.99	1.21

^a^ Caregivers were presented these statements and invited to rate their agreement on a five-point scale with 1 = strongly disagree and 5 = strongly agree. Means are presented.

## Data Availability

The raw data supporting the conclusions of this article will be made available by the authors on request.
